# The Effect of Anthelmintic Treatment on Coccidia Oocyst Shedding in a Wild Mammal Host with Intermittent Cestode Infection

**DOI:** 10.1155/2014/302903

**Published:** 2014-11-20

**Authors:** Radovan Václav, Jana Blažeková

**Affiliations:** Institute of Zoology, Slovak Academy of Sciences, Dúbravská Cesta 9, 845 06 Bratislava, Slovakia

## Abstract

While hosts are routinely exploited by a community of parasite species, the principles governing host responses towards parasites are unclear. Identifying the health outcomes of coinfections involving helminth macroparasites and microparasites is one area of importance for public and domestic animal health. For instance, it is controversial how deworming programmes affect incidence and severity of such important microparasite diseases as malaria. One problem is that most study systems involve domestic and laboratory animals with conditions hardly comparable to those of free-living animals. Here, we study the effect of anthelmintic treatment on coccidia infection intensity in wild Alpine marmots, *M. marmota*. Our results lend support to the hypothesis that helminth infection has a positive effect on concurrent microparasite infection. However, our work also points to the fact that within-host interactions between helminths and microparasites are context-dependent and can turn to negative ones once helminth burdens increase. Our study suggests that coccidia benefit from intermittent helminth infection in marmots due to the protective effects of helminth infection only during the early phase of the host's active season. Also, the marmot's response towards coccidia infection appears optimal only under no helminth infection when the host immune response towards coccidia would not be compromised, thereby pointing to the importance of regular intestinal helminth elimination by marmots just before hibernation.

## 1. Introduction

Under natural conditions, hosts are routinely exploited by a community of parasite species [1–3]. It is not surprising, therefore, that organisms evolved elaborate responses towards parasites under the selective pressure of multiple infection [1, 2, 4, 5]. As the complexity of host-parasite and parasite-parasite interactions in natural multiparasite disease systems is not sufficiently described, the principles governing these interactions and host responses towards parasites are far from clear [6, 7].

From an applied perspective, identifying the health outcomes of coinfections involving helminth macroparasites and microparasites such as protozoa, bacteria, or viruses is of utmost importance to public and domestic animal health officials [4, 8]. This is, on one hand, because macroparasite control has become widely available and efficient for human populations and domestic animals. On the other hand, before any such macroparasite control programme is incorporated in the fight against microparasites, its potential effects on the focal microparasites should be carefully taken into account. For example, despite intensive research, it is still controversial whether deworming in humans leads to decrease or increase in malaria incidence or severity [8].

Current theoretical and empiric work suggests that the outcome of coinfection can be different even for the same parasite system (e.g., [9]), depending on the level of resource competition between macro- and microparasites [4] or macroparasite burden and pathogenicity [2]. The optimal host immune response towards microparasites evolved not only under a bias of the immune response towards chronically infective macroparasites [10, 11], but also under fluctuating physiological states and parasite burdens [6]. Although host-parasite interactions have been intensively studied using domestic and laboratory animals [5, 12], it is questionable how relevant these studies are for understanding how free-living mammals respond to concurrent macro- and microparasite infection [6, 7, 13, 14].

Here, we study the effect of anthelmintic treatment on coccidia infection intensity in the free-living Alpine marmot,* M. marmota latirostris*. Alpine marmots are commonly coinfected with a tapeworm* Ctenotaenia marmotae* and coccidia* Eimeria* spp. [15–18]. While* Eimeria* spp. persist in marmots during hibernation,* C. marmotae* is expulsed by marmots before hibernation and reinfects them in the subsequent spring [16]. Our study has two goals. First, we determine the seasonal pattern in the infection intensity for the two commonest endoparasites of the Alpine marmot. Second, we test the hypotheses on the effect of helminth infection on microparasite infection intensity. If helminths affect microparasites via a reduction in the host's immune response [4], we predict that the coccidia oocyst shedding rate for marmots exposed to anthelmintic treatment should be lower than that for marmots from the control group. Alternatively, if helminths affect microparasites via a reduction in the host's resource levels [4], we predict that the coccidia oocyst shedding rate for marmots exposed to anthelmintic treatment should be higher than that for marmots from the control group.

## 2. Materials and Methods

### 2.1. Study System

We investigated the endoparasites of the Alpine marmot,* M. marmota latirostris*, in an alpine grassland of the Great Cold Valley in the High Tatra Mountains (49°10′N, 20°09′E), Slovakia, in 2011. The study site (ca. 200 ha) consists of about 16 territories (the number fluctuates between years) of a marmot subpopulation that is under investigation since 2010. The basic habitat features of the subpopulation studied are presented in [19]. The Alpine marmot is a true hibernating, territorial rodent (Rodentia: Sciuridae), living in social units of 2–20 individuals [20]. The active season of the Alpine marmots in the study population in the High Tatras spans from approximately mid-April to the beginning of October (Radovan Václav, unpublished data).

An anoplocephalid cestode* Ctenotaenia *(*Cittotaenia*)* marmotae* (Platyhelminthes: Cestoda) is the most prevalent and abundant helminth endoparasite of the Alpine marmot across its whole range, including Slovakia [15, 16, 18, 21–23]. Tapeworms are composed of successive reproductively viable segments, proglottids. After maturation, proglottids containing embryonated eggs detach from the tapeworm and are shed with host faeces. The weight of the cestode can reach more than 3% of a marmot's total body mass [17].* C. marmotae* is expulsed from the marmot gastrointestinal tract before hibernation in autumn and the parasite recolonizes its definite host in the subsequent spring [15, 16]. A variety of oribatid mite species act as intermediate hosts for the tapeworm [24] with marmots acquiring infection by accidentally ingesting infected mites [18].

Coccidia from the genus* Eimeria* are the most common microparasites of the Alpine marmot [15, 16]. In contrast to* C. marmotae*, they do not require intermediate hosts for transmission and persist in marmots even during hibernation [17]. Moreover, in contrast to* C. marmotae* for which the peak in the proglottid shedding rate takes place in late summer, the peak in the coccidia oocyst shedding rate occurs in early spring [17]. It is assumed that the number of oocysts discharged and the length of time they are shed depend on the number of sporulated oocysts in the initial infective dose (e.g., [25]). If the host becomes exposed again to the same coccidia, the host's protective immunity is responsible for a reduced oocysts shedding rate [25].

### 2.2. Experimental Design and Data Collection

In spring 2011 (late April-mid-May), we captured Alpine marmots in two-door, live-capture traps, following a procedure by Cohas et al. [26]. After capture, marmots were tranquillized with Zolétil 100 (0.10 mL/kg) and marked with a numbered ear tag and a transponder. In order to address the effect of helminth macroparasite infection on coccidia microparasites, each marmot was administered a broad-spectrum anthelmintic drug combination consisting of Ivermectinum and Praziquantelum. A broad-spectrum anthelmintic treatment was used to eliminate the potential effects of infection on coccidia not only by the most prevalent tapeworms but also by any helminth taxa known to infect Alpine marmots [18]. We administered 0.025 mL/kg of Ivermectinum (Biomectin, 10 mg/mL) and 0.1 mL/kg of Praziquantelum (Bancid, 56.8 mg/mL) intramuscularly to each marmot from the experimental group and 0.125 mL/kg of physiological solution intramuscularly to each marmot from the control group. The treatment was only applied once for each marmot. The effect of the treatment was examined considering all sampling units (individual faecal samples) at the level of marmot territories with four and three territories being assigned to the experimental (11 marmots) and control (13 marmots) groups, respectively.

From mid-May, we conducted intensive behavioural observations to confirm the structure of marmot social units within territories and started to collect fresh marmot faeces at seven territories where all (24) marmots were captured and either anthelmintic or physiological solution had been administered to them. Forty-five faecal samples were collected during field visits in May, July, and September at the specific defecation sites, latrines, which were used exclusively by territory members. For this study, we did not include faeces collected from marmot pastures because it was not possible to assign them unambiguously, due to territory overlaps, to individual territories. Also, juvenile marmots, which normally emerge during July, do not use latrines and defecate freely within territories (personal observations). Therefore, the faeces of untreated juveniles, which also can be classified visually based on their size, were not included in our analysis.

Fresh faecal samples were weighed to the nearest 0.1 g and stored in vials containing 2.5% (w/v) aqueous K_2_Cr_2_O_7_. The vials were stored at ambient temperature for five days to allow oocyst sporulation while the content of each vial was mixed thoroughly twice every day. After five days, the aqueous content of vials was extracted into clean vials and stored in a cool place and then in the fridge until examination (within 3 months). Oocysts and tapeworm eggs and proglottids were isolated by flotation in saturated sucrose solution and identified using oil immersion lenses on a compound microscope and Nomarski differential interference contrast microscopy. The oocyst numbers were quantified using the FLOTAC technique. Because most tapeworm proglottids containing eggs were crushed and did not allow exact quantification, the rate of tapeworm proglottid shedding was assigned to four levels at the scale from 0 to 3 based on the number of unambiguously identified* C. marmotae* proglottids. All coprological samples were analysed at the University of Veterinary and Pharmaceutical Sciences in Brno, the Czech Republic.

### 2.3. Data Analysis

The effect of anthelmintic treatment on* C. marmotae* and* Eimeria* spp. shedding rates was examined with general linear mixed models (GLMM). Both response variables,* C. marmotae* proglottid shedding rate and log-transformed* Eimeria* spp. oocyst numbers per gram (OPG) and faecal sample, were analysed assuming normally distributed errors. A visual inspection of model validation graphs (residuals versus fitted values and Q-Q plot) did not suggest a clear violation of the assumptions of homogeneity and normality for neither model [27]. Both models contained treatment, month, and the interaction between treatment and month as fixed factors. The tapeworm proglottid shedding rate was used as a covariate in the model on* Eimeria* spp. oocyst shedding. Because samples collected from the same territories are not statistically independent, territory identity was entered as a random factor. All tests were conducted with R software [28], using the lme4 package and the lmer function [29]. The Type III tests with denominator degrees of freedom were calculated based on a Satterthwaite's approximation and the least-square means were calculated using the lmerTest package [30]. The slice tests, examining the equality of simple effects of one factor for a given level of the other factor, were calculated using the lsmeans package and the glht function [31] with the *P* values reported being adjusted for multiple comparisons using the Bonferroni single-step procedure.

## 3. Results

### 3.1. Faecal Parasite Community Structure

Examining 45 marmot faecal samples collected from mid-May to the end of September at seven marmot territories, the presence of eggs or proglottids of a tapeworm* Ctenotaenia marmotae* (Platyhelminthes: Anoplocephalidae), the eggs of a nematode* Strongyloides* sp. (Nematoda: Strongyloididae), and the oocysts of coccidia* Eimeria* spp. (Apicomplexa: Eimeriidae) was detected in 58% (26/45: 2/12, 14/23, and 10/10 for May, July, and September), 9% (4/45: 1/12, 3/23, and 0/10 for May, July, and September), and 80% (36/45: 9/12, 20/23, and 7/10 for May, July, and September) samples, respectively.

### 3.2. Effect of Anthelmintic Treatment on Tapeworm Faecal Shedding

**Figure 1:**
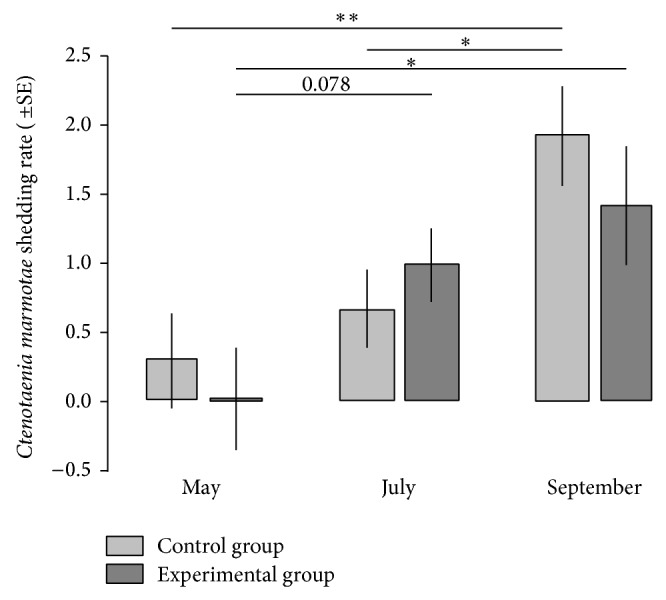
Effect of anthelmintic treatment on the faecal shedding rate of* Ctenotaenia marmotae* proglottids in the Alpine marmot,* M. marmota latirostris*. The* C. marmotae* shedding rate represents proglottid numbers on the scale from 0 to 3 per gram of a faecal sample, as more precise proglottid/egg quantification was not possible due to the physical damage of most proglottids. The symbols above lines ^∗∗^, ^∗^, and 0.078 refer to *P* < 0.001, *P* < 0.05, and *P* = 0.078, respectively. The *P* values reported are adjusted for multiple comparisons using the Bonferroni single-step procedure.

Considering the whole active season of marmots, anthelmintic treatment conducted shortly after hibernation did not significantly lower the total shedding rate of* Ctenotaenia marmotae* (effect of treatment: *F*_1, 4.64_ = 0.24, *P* = 0.644, effect of time of season: *F*_2, 38.78_ = 8.83, *P* < 0.001, and interaction between treatment and time of season: *F*_2, 38.87_ = 1.12, *P* = 0.333). Yet, examination of the interaction between experimental treatment and time of season revealed that anthelmintic treatment affected the dynamics of tapeworm faecal shedding during specific periods. Namely, the shedding rate increased from July to September but only for marmots in the control group (effect of time of season sliced by experimental treatment: difference between July and September for control group, *P* = 0.012; difference between July and September for experimental group, *P* = 0.637; Figure 1). Also, the proglottid shedding rate tended to increase from May to July but only for marmots in the experimental group (effect of time of season sliced by experimental treatment: difference between May and July for control group, *P* = 0.631; difference between May and July for experimental group, *P* = 0.078; Figure 1). That is to say, while the tapeworm shedding rate for the control group increased exponentially from May to September, the tapeworm shedding rate for the experimental group was sigmoidal, increasing abruptly from May to July and remaining high thereafter.

### 3.3. Effect of Anthelmintic Treatment on Coccidia Faecal Shedding

**Figure 2:**
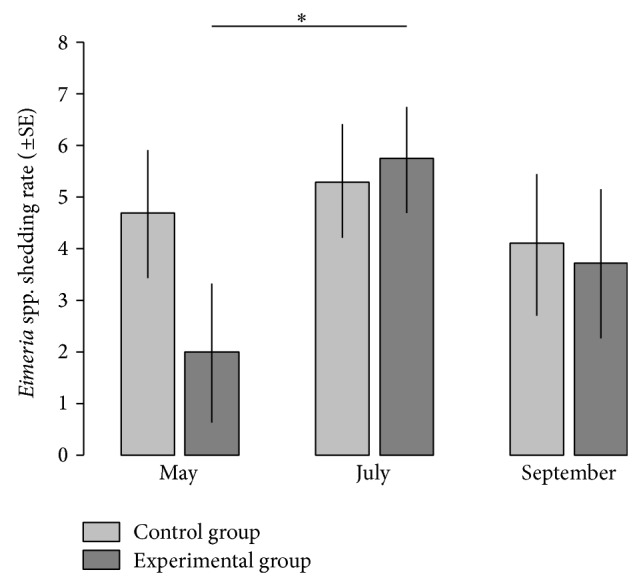
Effect of anthelmintic treatment on the faecal shedding rate of* Eimeria* spp. oocysts in the Alpine marmot,* M. marmota latirostris*. The* Eimeria* spp. shedding rate represents log-transformed oocyst numbers per gram of a faecal sample. The symbol above line ^∗^ refers to *P* < 0.05. The *P* value reported is adjusted for multiple comparisons using the Bonferroni single-step procedure.

While the shedding rate of tapeworm proglottids and* Eimeria* spp. oocysts was negatively associated (estimate ± SE = –1.02 ± 0.49, *t*_37.12_ = –2.07, *P* = 0.045), we did not find a significant difference in the shedding rate of oocysts between anthelmintic and control groups (effect of treatment: *F*_1, 5.62_ = 0.452, *P* = 0.528, effect of time of season: *F*_2, 36.46_ = 3.48, *P* = 0.041, and interaction between treatment and time of season: *F*_2, 37.51_ = 1.417, *P* = 0.255). Nevertheless, similarly, as for the dynamics of tapeworm shedding, we found that the shedding rate of* Eimeria* spp. oocysts increased from May to July but only for marmots in the experimental group (effect of time of season sliced by experimental treatment: difference between May and July for control group, *P* = 0.858; difference between May and July for experimental group, *P* = 0.036; Figure 2).

## 4. Discussion

Our experimental results suggest that helminth infection has a protective effect on the reproductive rates of coccidia microparasites in free-living Alpine marmots (hereafter referred to as marmots). Specifically, we found that when helminth infection was postponed in the beginning of the marmot active season via anthelmintic treatment, an increase in the shedding rate of* Eimeria* spp. oocysts also was postponed. Interestingly, a correlative result revealed a negative association between the infection intensity of a tapeworm macroparasite* C. marmotae* and the coccidia microparasites of the genus* Eimeria*, indicating that, under increased host exploitation, tapeworms negatively affect coccidia reproductive rates.

We stress that our deworming treatment affected only existing helminth infections because the shedding rates of tapeworm proglottids were comparable between experimental and control marmot groups from the mid active season (cf. [32]). Thus, our treatment can be considered as a postponement rather than prevention of helminth infection, and larger sample size would unlikely lead to revealing a significant effect of deworming treatment on tapeworm infection during the whole season. Importantly, this study shows that marmots of the study population in the High Tatras started to shed the proglottids of* C. marmotae* already in the second half of May. Assuming the prepatent period of* C. marmotae* to be 39–50 days [17], this study indicates that marmots can acquire infections with* C. marmotae* already during early April, that is, after hibernation but before emergence. This finding, suggesting that the first tapeworm infections take place in the underground burrow system, is important because the mechanism of host infections with Anoplocephalid tapeworms is still unclear [19].

Similar to previous studies conducted elsewhere [15–17], we found that* Eimeria* spp. prevalence is high in marmots in the High Tatras. However, compared to a previous study by Callait et al. [17], which revealed the peak in coccidia oocyst shedding during early spring, we found that the* Eimeria* spp. oocyst shedding rates in control marmots remained high through summer. It is possible that this difference is due to higher coccidia diversity in the High Tatras and a correspondingly longer time taking marmots to acquire resistance against this complex parasite group. In addition, this pattern can be due to a longer time taking marmots in the High Tatras to accumulate or sustain growing helminths, thereby allowing coccidia to reproduce for a longer time without elevated competition for host resources. The importance of coccidia diversity and seasonal changes in tapeworm burdens warrants further studies.

Our work lends support to the hypothesis [4] that helminth infection has a protective effect on concurrent microparasite infections via modulated host immunity. Nevertheless, our work also points to the fact that within-host interactions between helminths and microparasites can be context-dependent [2], and they can change to negative ones once helminth parasites mature and start to reproduce. This is feasible in our study system because both tapeworms* C. marmotae* and coccidia* Eimeria* spp. share the same body sections of their marmot hosts.

Based on our results, we suggest that coccidia benefit from intermittent helminth infection in marmots due to the protective effects of helminth infection during the first phase of the host active season. In turn, from the host perspective, this study indicates that marmot immune response towards continuous coccidia infection would be optimal only under no helminth infection when the host immune response is not compromised by a downregulated Th1-like response (e.g., [4, 5]). Even if this situation is hardly possible under natural conditions, we propose that intestinal worm expulsion by marmots, regularly taking place just before hibernation, might represent not only intestinal self-cure of worm infections [15, 17] but also an optimal strategy to control microparasite infection intensity during an important part of the host life cycle shortly after hibernation.

Coinfection with multiple parasites or parasite strains has an important bearing on parasite virulence because under competition parasites are thought to increase host resource exploitation [7]. Our study suggests that coccidia virulence might not be heightened in marmot populations if tapeworm infections are low or occur later in the season because competition between the two parasites groups is likely to be low (cf. [33]). In contrast,* Eimeria* virulence can be higher in seasons or marmot populations exhibiting higher worm burden or earlier worm infections when interspecific competition among parasites is likely to be elevated. Finally, interstrain competition also can affect parasite virulence in marmots because we detected a complex of distinct morphotypes of* Eimeria* spp. in faecal samples. A future work needs to establish whether specific* Eimeria* morphotypes prevail under different tapeworm burdens.

## 5. Conclusion

This experimental work demonstrates that correlational studies as well as those studies limiting their scope to macroparasite prevalence are not sufficient to depict the complexity of macroparasite-microparasite interactions. Our results suggest that the synergic effects of tapeworm infection on* Eimeria* spp. microparasites change into antagonistic ones under increased host exploitation by grown and reproducing macroparasites. We suggest that studying host-parasite and parasite-parasite interactions in free-ranging host species such as marmots, showing intermittent helminth infections, would be rewarding because this system mimics the one involving human populations receiving intermittent anthelmintic treatment while concurrently fighting microparasite diseases such as malaria.
